# Supplementary material for: Cryptic sequence features in the active postmortem transcriptome
Source: BMC Genomics. 2018 Sep 14;19:675. doi: 10.1186/s12864-018-5042-x (PMC6137749; doi:10.1186/s12864-018-5042-x)
Supplement: Supplementary file 1 — Proof that using the ‘Chaos Genome Representation’ method to extract mers from transcript sequences is more practical (computational efficient) than string-based search algorithms. (DOCX 20 kb) [file 12864_2018_5042_MOESM1_ESM.docx]

# **Proof: Chaos Game Representation is more efficient than string searching**

## Search for a substring ‘GATGC’ in a long string such as:

ATTCGCGCTGATGCGCTAGCTGAGCTAGTC

Let *m* be the length of a mer and *L* is the length of the gene. It takes *m* comparisons to match a substring. It takes *L-m*+1 matches to try. For *N* different mers, the total cost *S* is

S = *N***m**(*L-m*+1)

## **X-Y analysis, Chaos Game Representation (CGR)**

Before the search of a match, it takes an upfront cost to create *x,y* pairs of the chopped up string. Let *c* be the chunk size; it costs *c* to create an *x,y* pair for it. Therefore, the total upfront cost is *c**(*L*-*c*+1). The cost to create an *x,y* pair for a query mer is *m*. To find a match, the *x,y* pair of each mer must be compared with (*L*-*c*+1) of x-y pairs of chunks, hence the cost per mer is *m*+2*(*L*-*c*+1). The total cost for N mers is

Z= *c**(*L*-*c*+1)+N*(*m*+2*(*L*-*c*+1))

To compare performances, consider Z/S. If this quantity is less than 1, then the X-Y analysis outperforms string matching. For example, for N=1,000,000; L=10,0000, m=7:
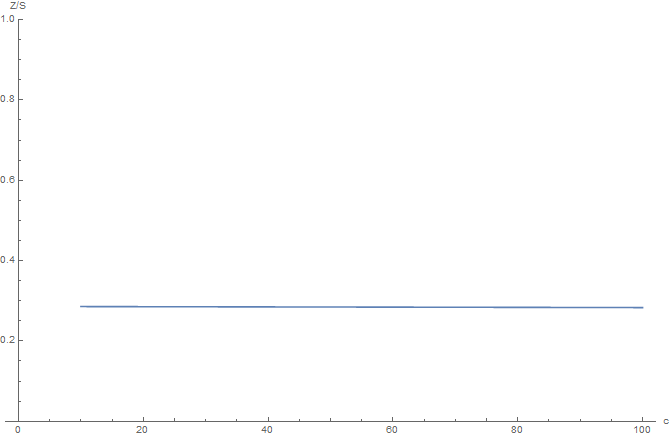


The graph indicates that the X-Y analysis is about 5 times faster than the plain string matching, virtually independent of the chunk size *c*.
